# National Trends in Utilization of Normothermic Machine Perfusion in DCD Liver Transplantation

**DOI:** 10.1097/TXD.0000000000001596

**Published:** 2024-04-09

**Authors:** Samir Abu-Gazala, Helen Tang, Peter Abt, Nadim Mahmud

**Affiliations:** 1 Department of Surgery, Perelman School of Medicine, University of Pennsylvania, Philadelphia, PA.; 2 Department of Medicine, Perelman School of Medicine, University of Pennsylvania, Philadelphia, PA.; 3 Division of Gastroenterology and Hepatology, Perelman School of Medicine, University of Pennsylvania, Philadelphia, PA.; 4 Department of Medicine, Corporal Michael J. Crescenz VA Medical Center, Philadelphia, PA.; 5 Leonard Davis Institute of Health Economics, University of Pennsylvania, Philadelphia, PA.; 6 Department of Biostatistics, Epidemiology and Informatics, Center for Clinical Epidemiology and Biostatistics, Perelman School of Medicine, University of Pennsylvania, Philadelphia, PA.

## Abstract

**Background.:**

In liver transplantation, advances in ex situ normothermic machine perfusion (NMP) have improved outcomes compared with traditional static cold storage (SCS) in donation after circulatory death (DCD) organs. We aimed to characterize trends in the utilization of NMP versus SCS in DCD liver transplantation in the United States.

**Methods.:**

This retrospective cohort study used data from the United Network for Organ Sharing database to identify recipient–donor adult liver transplant pairs from DCD donors from January 2016 to June 2022. Utilization of NMP and changes in donor risk index (DRI) and components between NMP and SCS were assessed across transplant year eras (2016–2018, 2019–2020, and 2021–2022). Statistical comparisons were made using the Kruskal-Wallis test or the chi-square test.

**Results.:**

A total of 3937 SCS and 127 NMP DCD donor transplants were included. Utilization of NMP ranged from ~0.4% to 3.5% from 2016 to 2021 and rose significantly to 11.2% in early 2022. Across transplant eras, median DRI increased significantly for SCS and NMP, but the magnitude of the increase was larger for NMP. With NMP DCDs, there were significant increases in median donor age, national share proportion, and “cold ischemic time” over time. Finally, there was a shift toward including higher DRI donors and higher model for end-stage liver disease score transplant recipients with NMP in later transplant eras.

**Conclusions.:**

In recent years, NMP utilization has increased and expanded to donors with higher DRI and recipients with higher model for end-stage liver disease score at transplant, suggesting increasing familiarity and risk tolerance with NMP technology. As NMP remains a relatively new technique, ongoing study of patient outcomes, organ allocation practices, and utilization patterns is critical.

Liver transplantation (LT) is a life-saving procedure for eligible patients with cirrhosis or liver malignancies, but the demand for organs exceeds availability. Thus, efforts to expand the number and viability of organs in the donor pool are critical.^1^ Among these, donation after circulatory death (DCD) liver donation has emerged as an important modality. Advances in ex situ normothermic machine perfusion (NMP) have helped decrease reperfusion-related complications of allograft dysfunction and ischemic cholangiopathy, mitigating some concerns associated with traditional static cold storage (SCS).^2-4^ In particular, a recent study by Ibeabuchi et al^3^ demonstrated that utilization of NMP for DCD donation was associated with a reduced hazard of graft failure and a lower incidence of need for retransplantation. As DCD outcomes continue to improve, DCDs are an increasing source of donor livers in the United States and Europe.^5^ However, to date, it remains unclear to what extent NMP utilization for DCD has expanded and whether the characteristics of recipients have evolved as experience with NMP accumulates. To address these knowledge gaps, this study aimed to characterize trends in the utilization of NMP versus SCS in DCD LT in the United States and to determine whether the use of NMP has expanded to higher-risk donor–recipient pairs over time.

## MATERIALS AND METHODS

### Study Design and Cohort Creation

This retrospective cohort study used de-identified data from the United Network for Organ Sharing (UNOS) transplant database. The University of Pennsylvania Institutional Review Board approved the research. Adult recipient–donor LT pairs from DCD donors were identified between January 2016 and June 2022. Patients receiving multiorgan transplants were excluded. Data on recipient demographic (age, sex, and race), causes of liver disease, transplant model for end-stage liver disease (MELD), transplant year, and donor characteristics comprising the donor risk index (DRI) were collected. DRI components included donor age, cause of death (anoxia, cerebrovascular accident, and other), donor race, regional versus national share, and cold ischemic time. Given that cold ischemia time as a component of DRI does not accurately reflect the perfused organ state during NMP, we also calculated the DRI excluding cold ischemia time. From the donor file, the classification of NMP versus SCS was ascertained following prior methods based on coded utilization of machine perfusion.^3^ Notably, we did not include patients who received hypothermic machine perfusion given that very few cases had been performed in the UNOS data set within the study window among adult DCD recipients (n = 5). Finally, center- and region-level data included distinct center codes, UNOS regions, and distances between donor and recipient hospitals.

### Statistical Analysis

Trends in utilization of NMP for DCD transplantation were plotted over time in terms of percent NMP utilization relative to all DCDs. For these data, the transplant year was divided into Q1/2 and Q3/4 for each year. Significant changes in secular trends were assessed using linear regression, where an era interaction variable was introduced on the basis of visual inspection. Beta coefficients and 95% confidence intervals were provided where relevant. Descriptive statistics for NMP versus SCS groups were presented as medians and interquartile ranges and frequencies and percentages for continuous and categorical variables, respectively. Statistical comparisons were made using the Wilcoxon rank-sum test and the chi-square or Fisher exact test, where indicated. Changes in DRI scores between NMP and SCS were assessed across transplant year eras (2016–2018, 2019–2020, and 2021–2022) using box plots, and medians were compared using Kruskal-Wallis tests. As a sensitivity analysis, this comparison was repeated using DRI, excluding cold ischemia time. A similar analytic approach was used to evaluate potential changes in distance between donor and recipient hospitals over transplant year eras, stratified by NMP versus SCS. Next, differences in individual DRI components for NMP DCDs across transplant year eras were evaluated using inferential statistics methods described previously. An alpha threshold of 5% for all tests was used to determine statistical significance. Finally, to visualize changes in donor–recipient “risk” over time, we plotted DRI against transplant MELD in each transplant era, separately for SCS and NMP. Differences were assessed qualitatively in these plots as an exploratory analysis. All analyses were performed using STATA version 17.0/BE (College Station, TX).

## RESULTS

**FIGURE 1.**
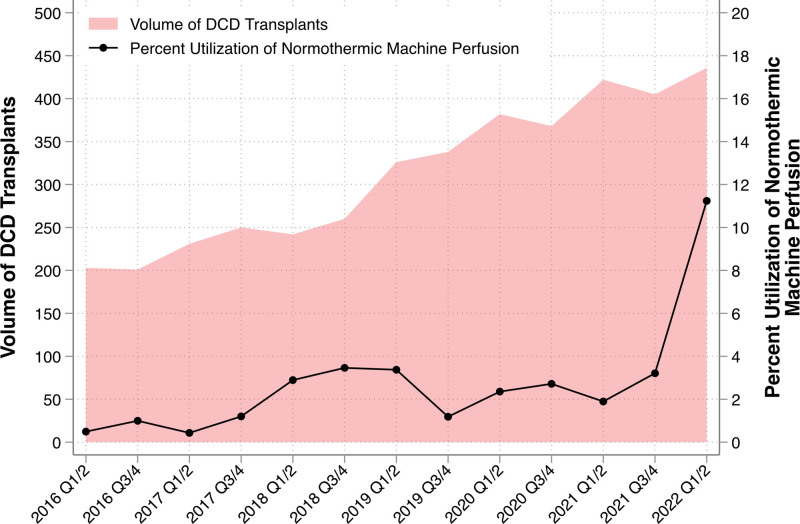
Trends in utilization of normothermic machine perfusion in DCD liver transplantation. DCD, donation after circulatory death.

**FIGURE 2.**
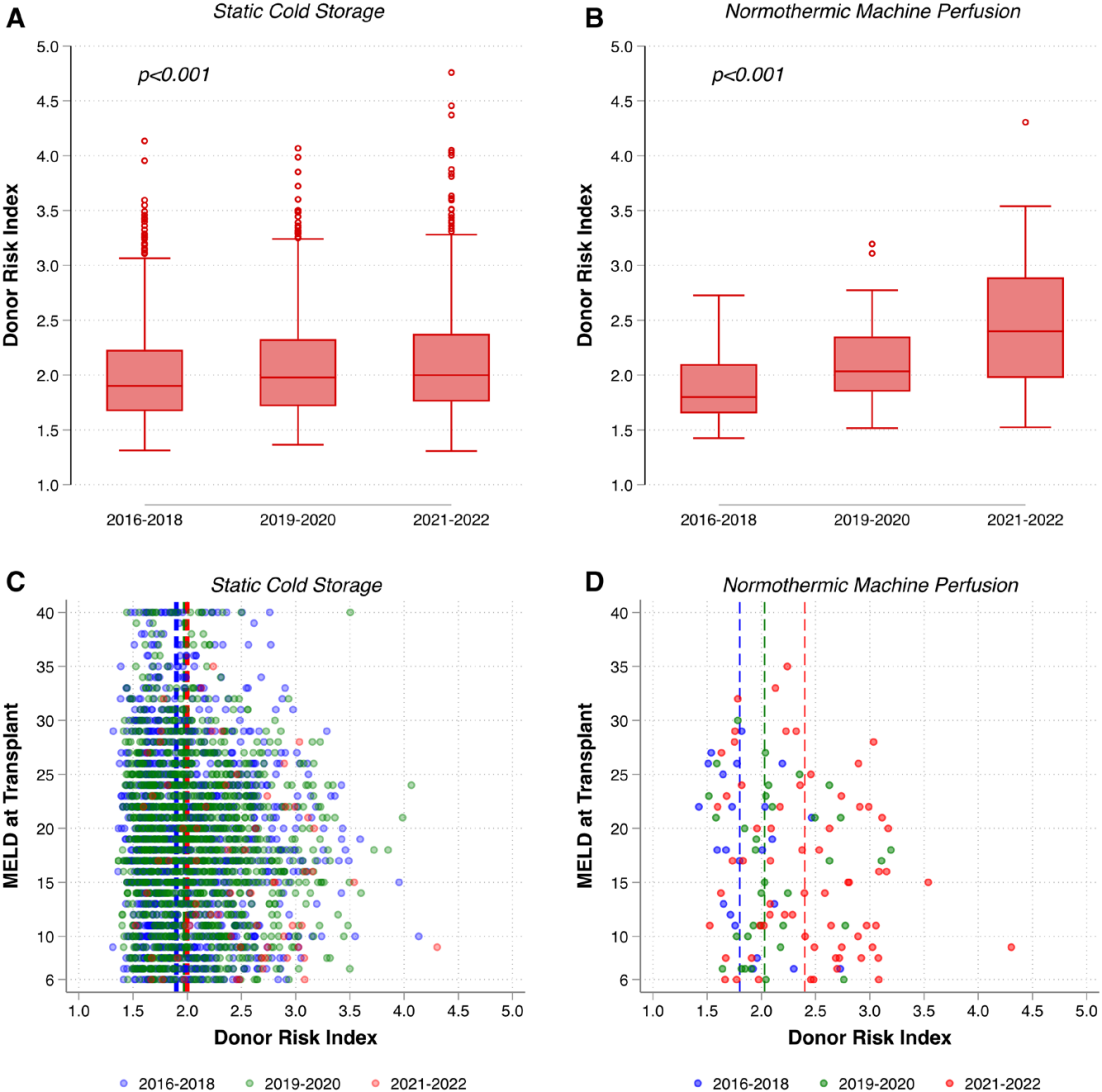
Donor risk index by era and MELD at transplant for static cold storage and normothermic machine perfusion. Box plots of DRI distribution (A and B). Over time, scatterplots of donor risk index and MELD at transplant were stratified by preservation method (C and D). Vertical dashed lines in panels C and D correspond to median values for the transplant era. MELD, model for end-stage liver disease.

After the application of selection criteria, a total of 4064 DCD recipient–donor transplant pairs were included in the analytic cohort, of whom 3937 (96.9%) were SCS and 127 (3.1%) were NMP DCD donors. A total of 104 distinct centers performed DCD transplants in the study window, of which 25 centers (24.0%) used NMP. Among UNOS regions, the proportion of DCD transplants using NMP varied substantially (*P* < 0.001; **Table S1, SDC,** http://links.lww.com/TXD/A639); the proportion was highest in region 1 (20.9%) and lowest in region 2 (0.4%). In evaluating temporal trends, the percent utilization of NMP with DCD transplantation ranged from ~0.4% to 3.5% from 2016 to 2021; this rose significantly to 11.2% in the first half of 2022 (Figure 1). There was an associated break of linear trend between 2021 Q3/4 and 2022 Q3/4, such that the rate of percent NMP change increased from 0.18% (95% CI, –0.03% to 0.40%) to 8.03% (95% CI, 4.87%–11.2%; interaction *P* < 0.001). Overall, recipients of NMP DCD transplants were younger (median 62 versus 59 y, *P* = 0.02), had lower MELD at transplant (median 17 versus 18, *P* = 0.007), and received higher DRI score organs (median 2.1 versus 2.0, *P* < 0.001; Table 1) compared with recipients of SCS transplants. Looking at specific DRI components between NMP and SCS, NMP DCD transplant recipients had higher median donor age (43 versus 37 y, *P* < 0.001) and longer recorded “cold ischemic time” (median 8.8 versus 5.3 h, *P* < 0.001). Across transplant eras, there were significant increases in DRI for both SCS and NMP (*P* < 0.001); however, the magnitude of increase from 2016–2018 to 2021–2022 was larger for NMP (ie, 1.8–2.4 for NMP versus 1.9–2.0 for SCS; Figure 2A and B); findings were similar when the cold ischemic time was excluded from DRI calculation (**Figure S1, SDC,** http://links.lww.com/TXD/A639). Although the distance between donor and recipient hospitals also increased over transplant year eras for both SCS (*P* = 0.02) and NMP (*P* < 0.001), the degree of increase was substantially greater for NMP DCD transplantations (**Figure S2, SDC,** http://links.lww.com/TXD/A639). For example, in 2021–2022, the median distance for NMP was 138.5 miles versus 26.0 miles in 2016–2018, whereas for SCS, the median distance in these periods was 88.0 and 66.0 miles, respectively. Over this period for NMP DCDs, there was a significant increase in donor age (median 46 y in 2021–2022 versus 27 y in 2016–2018, *P* = 0.004), national share proportion (31.4% in 2021–2022 versus 0.0% in 2016–2018, *P* < 0.001), and “cold ischemic time” (median 9.9 h in 2021–2022 versus 7.2 h in 2016–2018, *P* < 0.001; **Table S2, SDC,** http://links.lww.com/TXD/A639). Finally, in plots of DRI versus transplant MELD by era, there was a shift toward including higher DRI donors and higher transplant MELD recipients with NMP in later transplant eras (Figure 2C and D).

**TABLE 1.** Cohort characteristics for DCD liver transplantation, stratified by preservation method, and differences in donor risk index components

Factor	Static cold storage (N = 3937)	Normothermic machine perfusion (N = 127)	*P*
Recipient age, median (IQR)	59 (52–65)	62 (54–66)	0.02
Sex, n (%)			0.63
Female	1254 (31.9)	43 (33.9)	
Male	2683 (68.1)	84 (66.1)	
Race, n (%)			0.82
White	3004 (76.3)	100 (78.7)	
Black	206 (5.2)	4 (3.1)	
Hispanic	534 (13.6)	19 (15.0)	
Asian	122 (3.1)	3 (2.4)	
Other	71 (1.8)	1 (0.8)	
Cause of liver disease, n (%)			0.32
Hepatitis C virus	761 (19.3)	20 (15.7)	
Alcohol	1253 (31.8)	42 (33.1)	
Hepatitis B virus	101 (2.6)	5 (3.9)	
NASH/cryptogenic	1147 (29.1)	33 (26.0)	
Primary sclerosing cholangitis	128 (3.3)	3 (2.4)	
Autoimmune	112 (2.8)	3 (2.4)	
Other	326 (8.3)	13 (10.2)	
Primary biliary cholangitis	109 (2.8)	8 (6.3)	
MELD at transplant, median (IQR)	18 (13–24)	17 (10–23)	0.007
Transplant year, n (%)			<0.001
2016	401 (10.2)	3 (2.4)	
2017	477 (12.1)	4 (3.1)	
2018	486 (12.3)	16 (12.6)	
2019	649 (16.5)	15 (11.8)	
2020	731 (18.6)	19 (15.0)	
2021	806 (20.5)	21 (16.5)	
2022	387 (9.8)	49 (38.6)	
Donor risk index, median (IQR)	1.96 (1.71–2.31)	2.10 (1.82–2.64)	<0.001
Donor risk index excluding cold ischemia time, median (IQR)	1.85 (1.62–2.18)	1.95 (1.68–2.37)	0.01
**Donor risk index components**	**Static cold storage (N = 3937**)	**Normothermic machine perfusion (N = 127**)	***P***
Donor age, median (IQR)	37 (27–48)	43 (32–53)	<0.001
Donor age, y, n (%)			
40–50	853 (21.7)	31 (24.4)	0.46
50–60	718 (18.2)	34 (26.8)	0.02
60–70	133 (3.4)	8 (6.3)	0.08
≥70	1 (<1)	0 (0.0)	1.00
Cause of death, n (%)			
Anoxia	2091 (53.1)	73 (57.5)	0.33
Cerebrovascular accident	655 (16.6)	28 (22.0)	0.11
Other	2 (0.1)	0 (0.0)	1.00
Race, n (%)			
Black	396 (10.1)	11 (8.7)	0.61
Other	74 (1.9)	1 (0.8)	0.73
Regional share	1086 (27.6%)	26 (20.5%)	0.08
National share	545 (13.8%)	24 (18.9%)	0.11
Cold ischemic time, h, median (IQR)	5.3 (4.4, 6.3)	8.8 (6.5, 10.9)	<0.001

DCD, donation after circulatory death; IQR, interquartile range; MELD, model for end-stage liver disease; NASH, nonalcoholic steatohepatitis.

## DISCUSSION

In the United States, DCD donation has expanded the donor pool for LT. Still, DCD organs remain generally less desirable than donation after brain death organs.^6^ NMP technology has improved outcomes associated with DCD donation and now represents an increasing proportion of DCD donation,^4,7^ although to date, only about one-quarter of DCD-performing centers have used NMP technology. In this retrospective UNOS study of DCD LT from 2016 to 2022, we found that the utilization of NMP technology has increased over time, from 0.5% of all DCD transplants in 2016 to 11.2% in 2022; there was a particularly sharp increase in utilization moving from 2021 to 2022. In the early transplant era, NMP was used in younger donors and never with national sharing. However, over time, the median donor age with NMP increased by nearly 2 decades, with a significantly longer distance between donor and recipient hospitals, and a substantial proportion was shared across UNOS regions or nationally. This is reflected in increased “cold ischemic time” with NMP transplants in the most recent transplant era (2021–2022). These variables are all drivers of the significantly increased DRI over time that was observed with NMP utilization. Additionally, in the 2021–2022 transplant era, NMP DCD organs were transplanted for the first time into recipients with MELD scores >30. These findings are strong signals that transplant centers have become more familiar with NMP technology and that they are increasingly willing to use NMP with higher-risk donors and sicker recipients. Taken together with prior work demonstrating a potential reduction in posttransplant graft failure with NMP DCD transplantation,^3^ the adoption of machine perfusion technology is likely to continue to expand. Based on the observed trends, it will be important for future research to evaluate potential differences in posttransplant outcomes among sicker recipients who have received NMP DCD transplantation.

This study has several limitations that we acknowledge in this study. First, an important limitation of the UNOS data set is its definition of cold ischemic time, which is associated with poor graft outcomes.^8,9^ However, cold ischemic time traditionally refers to the time an organ is outside of the donor body before transplantation and, therefore, includes NMP time, which causes less physiological stress on the graft than the equivalent period for SCS livers. As such, increased reported cold ischemic time could be driving observed increases in DRI associated with NMP without accurately reflecting true donor organ risk (although, as noted previously, median donor age also increased). Nonetheless, increased “cold ischemic time” with higher proportions of regional and national sharing over time suggests that NMP enables the viability of transporting DCD organs over longer distances. As utilization of NMP continues to grow over time, it is imperative that national databases, including UNOS, include variables that capture elements specific to NMP protocols. Second, there is also possible misclassification of exposures with regard to NMP utilization; however, there are specified fields in the UNOS database to classify the use of machine perfusion technology with DCD transplantation, and thus, the impact of this potential bias is likely minimal. Third, we did not analyze hypothermic machine perfusion, given that very few cases had been documented in the UNOS data set in the study window. There are ongoing active clinical trials that will make this an important area of future study. Finally, due to the limited granularity of data in the UNOS data set, we were not able to reliably differentiate normothermic regional perfusion (in situ) from ex situ NMP, and thus, both were classified as “NMP.” As noted previously, this is another area where additional variables are needed in the UNOS registry, given that the process variables are fundamentally distinct between different machine perfusion techniques. For example, normothermic regional perfusion involves rapid in situ machine perfusion followed by cross-clamp and cold ischemia time, whereas ex situ NMP entails brief periods of cold ischemia time on either end of an extended machine perfusion time.

In conclusion, our study reports an increased utilization of NMP in recent years, and NMP has been used in donors with higher DRI and recipients with higher MELD at transplant over time. This suggests increasing familiarity and growing risk tolerance with NMP technology. However, because NMP remains a relatively new technique, ongoing review of patient outcomes, organ allocation practices, and utilization patterns are critical areas for future study.

## Supplementary Material


