# Supplementary material for: National Trends in Utilization of Normothermic Machine Perfusion in DCD Liver Transplantation
Source: Transplant Direct. 2024 Apr 9;10(5):e1596. doi: 10.1097/TXD.0000000000001596 (PMC11005893; doi:10.1097/TXD.0000000000001596)

Table S1 – Distribution of NMP versus SCS DCD Transplantations Performed by UNOS Region (row percentages shown)*

UNOS Region	Static Cold Storage<br>N = 3937	Normothermic Machine Perfusion<br>N = 127	Total
1	72 (79.1%)	19 (20.9%)	**91**
2	248 (99.6%)	1 (0.4%)	**249**
3	661 (98.8%)	8 (1.2%)	**669**
4	331 (95.1%)	17 (4.9%)	**348**
5	813 (96.3%)	31 (3.7%)	**844**
6	120 (99.2%)	1 (0.8%)	**121**
7	376 (97.7%)	9 (2.3%)	**385**
8	218 (96.9%)	7 (3.1%)	**225**
9	132 (95.7%)	6 (4.3%)	**138**
10	650 (97.0%)	20 (3.0%)	**670**
11	316 (97.5%)	8 (2.5%)	**324**
**Total**	**3,937**	**127**	**4,064**

** p-value for association between SCS vs. NMP and UNOS Region <0.001*

Table S2 – Changes in DRI Components of Time with Normothermic Machine Perfusion

Factor	2016-2018<br>N = 23	2019-2020<br>N = 34	2021-2022<br>N = 70	p-value
Donor Age, median (IQR)	27 (22, 47)	41.5 (32, 50)	46 (34, 53)	**0.004**
Donor Age 40-50	4 (17.4%)	13 (38.2%)	14 (20.0%)	0.10
Donor Age 50-60	3 (13.0%)	8 (23.5%)	23 (32.9%)	0.17
Donor Age 60-70	1 (4.3%)	1 (2.9%)	6 (8.6%)	0.61
Donor Age ≥70	0 (0.0%)	0 (0.0%)	0 (0.0%)	-
Cause of Death = Anoxia	15 (65.2%)	17 (50.0%)	41 (58.6%)	0.52
Cause of Death = CVA	2 (8.7%)	10 (29.4%)	16 (22.9%)	0.16
Cause of Death = Other	0 (0.0%)	0 (0.0%)	0 (0.0%)	-
Race = Black	2 (8.7%)	4 (11.8%)	5 (7.1%)	0.76
Race = Other	1 (4.3%)	0 (0.0%)	0 (0.0%)	0.18
Regional Share	2 (8.7%)	5 (14.7%)	19 (27.1%)	0.11
National Share	0 (0.0%)	2 (5.9%)	22 (31.4%)	**<0.001**
Cold Ischemic Time (hours), median (IQR)	7.2 (5.6, 8.5)	7.6 (5.5, 9.3)	9.9 (8.3, 12.6)	**<0.001**

Figure S1 – Box Plots of DRI Distribution (A/B) Over Time with Cold Ischemia Time Excluded from DRI Calculation, Stratified by Preservation Method

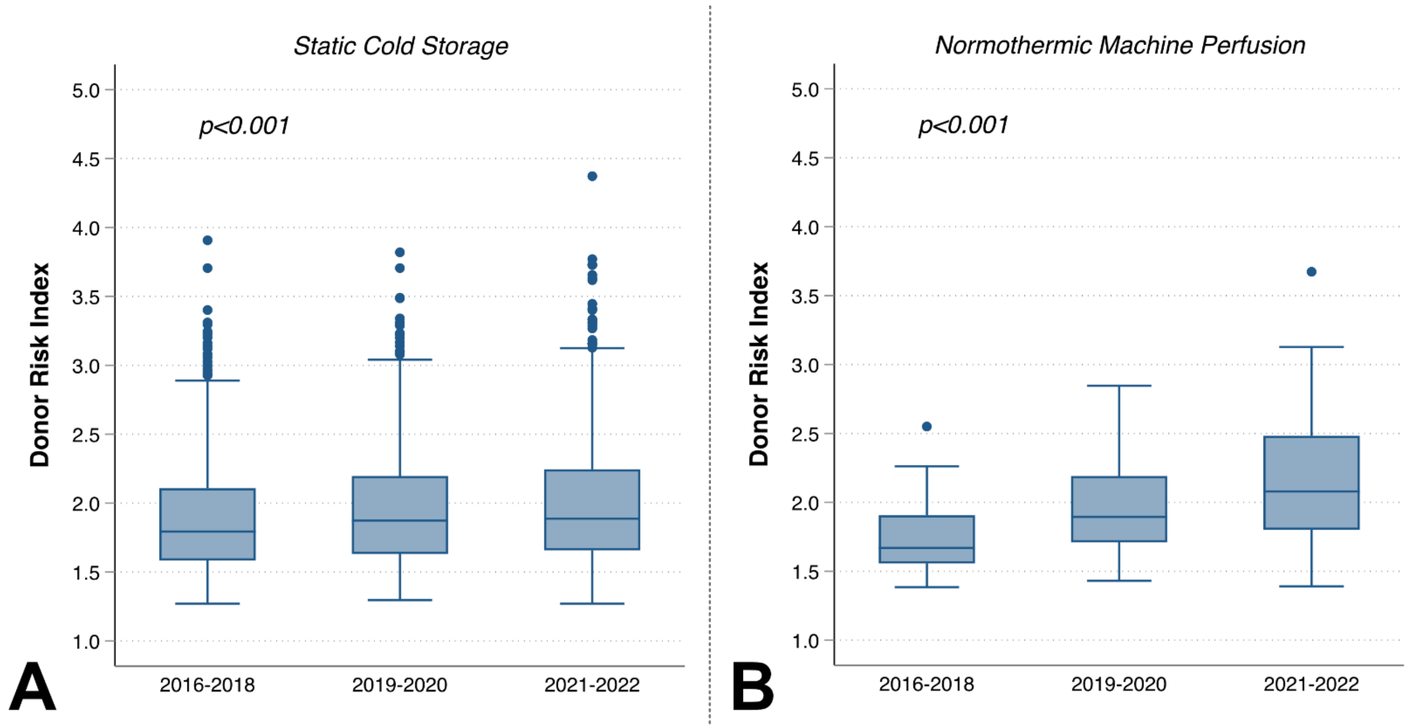

Figure S2 – Box Plots of Distance between Donor and Recipient Hospital (A/B) Over Time (in miles), Stratified by Preservation Method

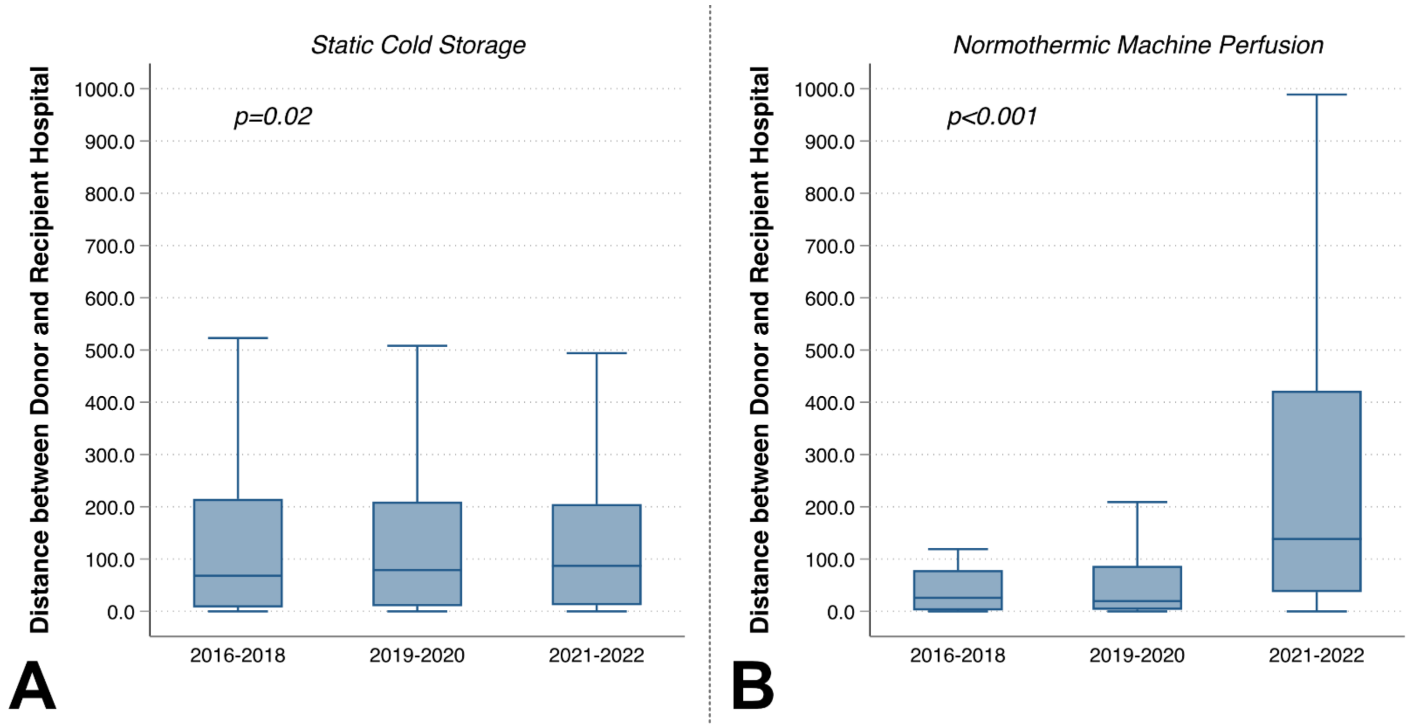

Supplement: Supplementary file 1 [file txd-10-e1596-s001.pdf]
